# Supplementary material for: Complex‐centric proteome profiling by SEC‐SWATH‐MS
Source: Mol Syst Biol. 2019 Jan 14;15(1):e8438. doi: 10.15252/msb.20188438 (PMC6346213; doi:10.15252/msb.20188438)
Supplement: Supplementary file 8 — Dataset EV7 [file MSB-15-e8438-s008.zip › feature_plots_string/Q71UM5.pdf]

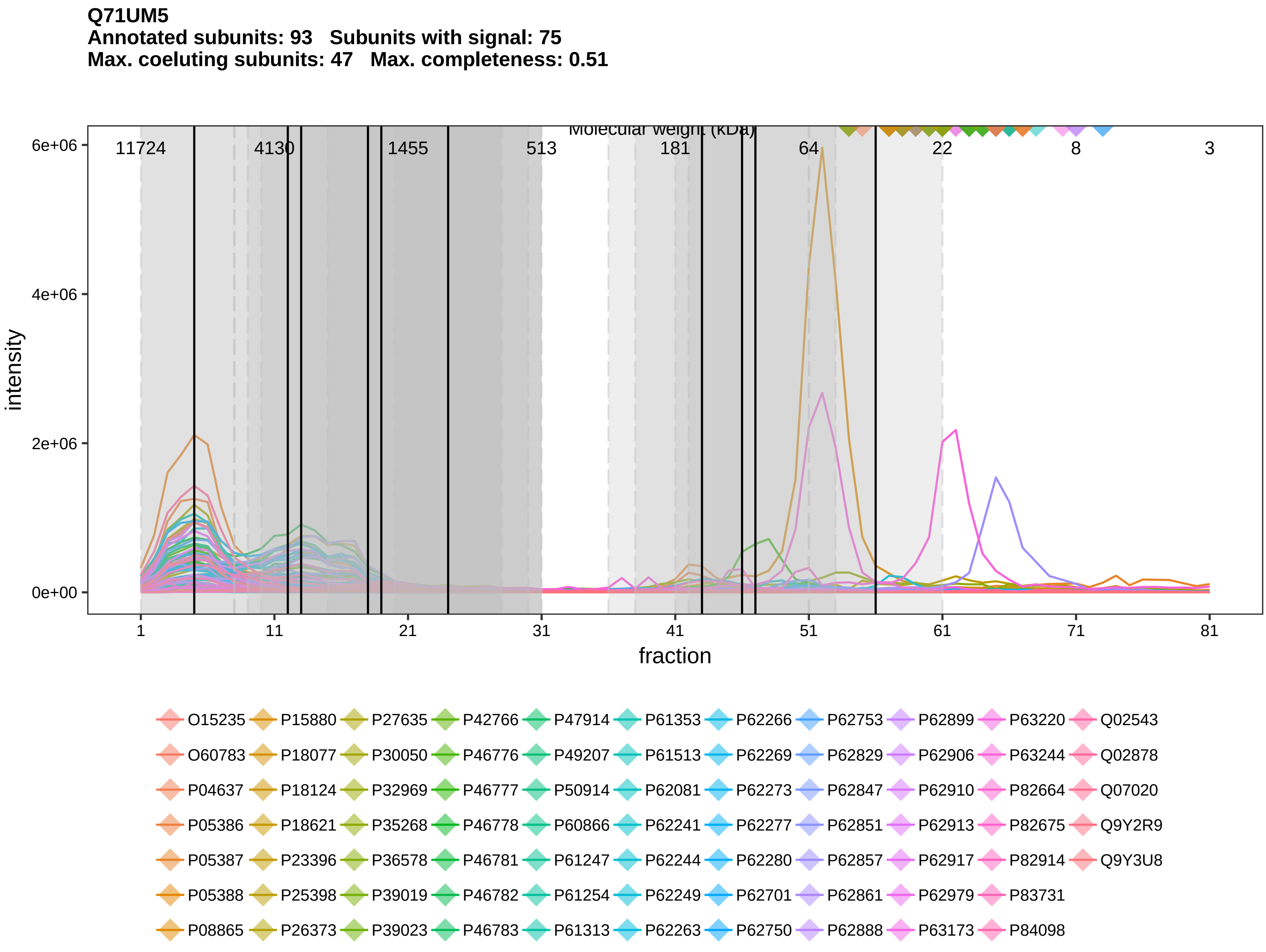
Q71UM5
Annotated subunits: 93 Subunits with signal: 75
Max. coeluting subunits: 47 Max. completeness: 0.51
Molecular weight (kDa)
11724
4130
1455
513
181
64
22
8
3
6e+06
4e+06
2e+06
0e+00
intensity
1
11
21
31
41
51
61
71
81
fraction
O15235 P15880 P27635 P42766 P47914 P61353 P62266 P62753 P62899 P63220 Q02543
O60783 P18077 P30050 P46776 P49207 P61513 P62269 P62829 P62906 P63244 Q02878
P04637 P18124 P32969 P46777 P50914 P62081 P62273 P62847 P62910 P82664 Q07020
P05386 P18621 P35268 P46778 P60866 P62241 P62277 P62851 P62913 P82675 Q9Y2R9
P05387 P23396 P36578 P46781 P61247 P62244 P62280 P62857 P62917 P82914 Q9Y3U8
P05388 P25398 P39019 P46782 P61254 P62249 P62701 P62861 P62979 P83731
P08865 P26373 P39023 P46783 P61313 P62263 P62750 P62888 P63173 P84098
